# Interleukin 21 Signaling in B Cells Is Required for Efficient Establishment of Murine Gammaherpesvirus Latency

**DOI:** 10.1371/journal.ppat.1004831

**Published:** 2015-04-13

**Authors:** Christopher M. Collins, Samuel H. Speck

**Affiliations:** 1 Emory Vaccine Center, Emory University School of Medicine, Atlanta, Georgia, United States of America; 2 Department of Microbiology and Immunology, Emory University School of Medicine, Atlanta, Georgia, United States of America; University of Wisconsin-Madison, UNITED STATES

## Abstract

The human gammaherpesviruses take advantage of normal B cell differentiation pathways to establish life-long infection in memory B cells. Murine gammaherpesvirus 68 (MHV68) infection of laboratory strains of mice also leads to life-long infection in memory B cells. To gain access to the memory B cell population, MHV68 infected B cells pass through the germinal center reaction during the onset of latency and require signals from T follicular helper (T_FH_) cells for proliferation. Interleukin 21 (IL-21), one of the secreted factors produced by T_FH_ cells, plays an important role in both the maintenance of the germinal center response as well as in the generation of long-lived plasma cells. Using IL-21R deficient mice, we show that IL-21 signaling is required for efficient establishment of MHV68 infection. In the absence of IL-21 signaling, fewer infected splenocytes are able to gain access to either the germinal center B cell population or the plasma cell population – the latter being a major site of MHV68 reactivation. Furthermore, the germinal center B cell population in IL-21R^-/-^ mice is skewed towards the non-proliferating centrocyte phenotype, resulting in reduced expansion of infected B cells. Additionally, the reduced frequency of infected plasma cells results in a significant reduction in the frequency of splenocytes capable of reactivating virus. This defect in establishment of MHV68 infection is intrinsic to B cells, as MHV68 preferentially establishes infection in IL-21R sufficient B cells in mixed bone marrow chimeric mice. Taken together, these data indicate that IL-21 signaling plays multiple roles during establishment of MHV68 infection, and identify IL-21 as a critical T_FH_ cell-derived factor for efficient establishment of gammaherpesvirus B cell latency.

## Author Summary

Gammaherpesviruses establish life-long infection in B cells by taking advantage of the host immune response that is generated during primary infection. During initial infection, the immune system responds by inducing rapid proliferation of responding B cells during the germinal center reaction. This response is highly coordinated and relies on the interplay of multiple cell types. CD4 T helper cells are an important component of the germinal center reaction in that they communicate with B cells by providing both proliferation and survival signals. Gammaherpesviruses infect B cells that receive these signals, resulting in proliferation and survival of infected cells, allowing the virus to establish life-long infection. Here we show that interleukin 21 (IL-21), one of the signaling factors produced by CD4 T cells, is required for efficient establishment of infection in a mouse model of gammaherpesvirus infection. In the absence of IL-21 signaling, the viral load is markedly reduced and the composition of the infected cell population is altered to cell types that are less proliferative and produce less virus. These results demonstrate how gammaherpesviruses are able to take advantage of the immune response being generated against it to establish lifelong infection.

## Introduction

The human gammaherpesviruses, Epstein-Barr virus (EBV) and Human herpesvirus 8 (HHV-8 also known as Kaposi’s sarcoma associated herpesvirus or KSHV), are B cell tropic viruses that establish life-long infection in memory B cells, which provide a quiescent, long-lived reservoir for the virus to remain latent in. To gain access to the memory pool, these viruses must pass through the germinal center reaction. The role of EBV in manipulating B cell biology to drive infected B cells through the germinal center reaction has been well established (reviewed in [1]). EBV encodes proteins that mimic signals involved in driving B cells through the germinal center reaction. LMP-1 is a membrane protein the mimics CD40 signaling [2], whereas LMP2A mimics tonic BCR signaling [3]. Primary infection with HHV-8 is not as well understood, and what role the virus plays in manipulating infected B cells to gain access to the memory pool is not known. Infection of laboratory strains of mice with the closely related Murine gammaherpesvirus 68 (MHV68), a small animal model of gammaherpesvirus pathogenesis, has also been shown to lead to infection of germinal center B cells at the peak of latency and establishment of life-long infection in memory B cells [4–8]. We have recently shown that MHV68 requires signals from T follicular helper (T_FH_) cells for expansion of infected germinal center B cells during the onset of latency [9]. However, these experiments were performed in the context of nearly complete ablation of T_FH_ cell help and germinal center formation. Because of this, it remains unclear whether or not MHV68 plays an active role in this process by by-passing specific signals received from T_FH_ cells to influence the fate of infected B cells, or if the virus plays a more passive role, relying instead on normal germinal center B cell biology for passage through the germinal center reaction.

During a T cell-dependent immune response, antigen activated B cells present antigen to primed cognate CD4 T cells at the border of the T cell and B cell zones [10]. These B cells then can undergo one of two fates. Some migrate to the extra-follicular space where they differentiate into short-lived plasma cells that produce low affinity antibodies and survive for only a few days [11]. Others enter the B cell follicle where they initiate a germinal center reaction [10]. During the germinal center reaction, B cells undergo repetitive cycles of clonal proliferation and somatic hypermutation in the dark zone of the germinal center followed by selection in the light zone [12]. After several cell divisions in the dark zone, germinal center B cells migrate to the light zone, where they take up antigen from follicular dendritic cells and present it to T_FH_ cells in order to receive survival signals [12]. These B cells then either re-enter the dark zone, where they undergo more proliferation and somatic hypermutation, or they exit the germinal center reaction and differentiate into memory B cells or long-lived plasma cells [13]. Germinal center B cells with the highest affinity BCRs are able to capture more antigen in the light zone and outcompete B cells with lower affinity BCRs for T_FH_ cell help. The amount of antigen captured by B cells in the light zone directly correlates with the amount of cell division and somatic hypermutation, resulting in selection of cells with the highest affinity for antigen [14].

T_FH_ cells deliver survival signals to B cells by expressing multiple surface markers that bind ligands on germinal center B cells, as well as by producing soluble factors. One of the major secreted factors T_FH_ cells produce is interleukin 21 (IL-21) [15–17]. Because T_FH_ cells express the IL-21 receptor [15,17], several early reports indicated that IL-21 signaling in CD4 T cells plays a role in the differentiation of T_FH_ cells [16,18]. However, more recent reports have shown that IL-21 is not required for generation of T_FH_ cells, and that the role of IL-21 signaling in the humoral response is intrinsic to B cells [19–21]. IL-21 is a pleiotropic cytokine that has multiple effects on B cells depending on context [22]. During a T-dependent immune response, IL-21 affects B cell biology at multiple levels. While IL-21 is not required for the extra-follicular plasma cell response that produces short-lived plasma cells, it has been shown to be critical in generating long-lived plasma cells and high affinity antibodies [19,23]. Since IL21R signals through STAT3 [24,25], this differential requirement for IL-21 in generating plasma cells is likely due to the requirement for STAT3 in generating post-germinal center derived plasma cells, but not for short-lived extra-follicular plasma cells [26]. IL-21 signaling in B cells up-regulates Bcl-6 expression [20,27,28], a transcriptional regulator that is essential for differentiation of germinal center B cells [29]. Mice defective in IL-21 signaling are capable of generating germinal centers, however their maintenance is impaired [20,23], and germinal center B cells in these mice are less proliferative and undergo less somatic hypermutation [21].

These two roles for IL-21 signaling are notable for MHV68. We have previously shown that differentiation of latently infected B cells into plasma cells is linked to viral reactivation [30], which appears to be a conserved strategy among the gammaherpesviruses [31–35]. We have also shown that the magnitude of the germinal center response correlates with the level of infection and that MVH68 infected B cells rely on signals from T_FH_ cells for proliferation [9]. Because of this, we hypothesized that IL-21 plays a major role in promoting establishment of MHV68 infection. To delineate the role of IL-21 signaling in MHV68 infection, we characterized infection in IL-21R^-/-^ mice. We show that IL-21 signaling in B cells is critical for efficient establishment of MHV68 infection and is required for efficient access to both the germinal center and plasma cell fractions. The reduced frequency of infected plasma cells results in a significant reduction in the frequency of infected splenocytes that are capable of reactivating virus. Notably, although there is a statistically significant defect in generating germinal center B cells in IL-21R^-/-^ mice, it is much smaller than the defect in establishment of MHV68 infection, indicating that the lack IL-21 signaling has a greater effect on infected B cells than on uninfected B cells. Both infected and uninfected germinal center B cell populations in IL-21R^-/-^ mice are skewed towards the non-proliferating centrocyte pool, resulting in reduced proliferation of infected germinal center B cells and reduced infection. Taken together, our data identifies IL-21 as a T_FH_ cell derived factor that is critical for efficient establishment of gammaherpesvirus latency, and that it functions at multiple levels during the onset of latency.

## Results

### Il-21 signaling is required for efficient establishment of viral latency

**Fig 1:**
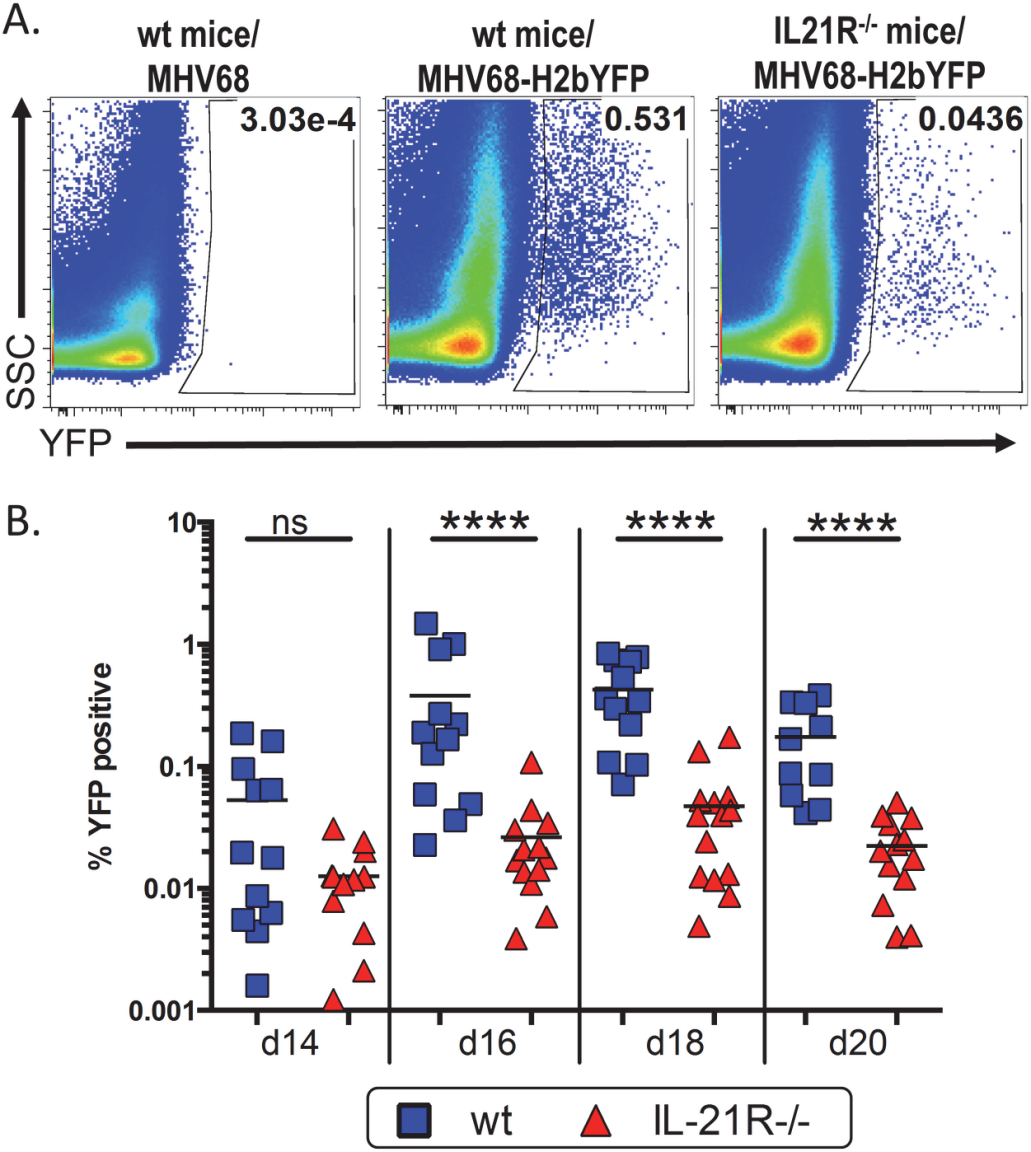
IL-21 signaling is required for efficient establishment of MHV68 latency. Mice were infected intranasally with 1,000 pfu of MHV68-H2bYFP and splenocytes were harvested at the indicated time points. (A) Representative flow plots showing identification of YFP^+^ B cells at d16 post-infection. Gates were drawn based on the distribution of splenocytes from wt mice infected with wt MHV68 to distinguish YFP^+^ cells from background fluorescence. Flow plots were gated on CD3^-^, CD4^-^, CD8^-^, B220^+^ cells. (B) Frequency of YFP^+^ B cells. Results are compiled from 3 independent experiments at each time point with 3–6 mice per group. Each symbol represents an individual mouse, and the horizontal lines represent the mean frequency of infected B cells. ns = not significant; ****, p<0.0001.

To determine what role Il-21 signaling plays in establishment of MHV-68 infection, we infected IL-21R^-/-^ mice with MHV68-H2bYFP, a previously described transgenic virus [5] that expresses a fusion protein consisting of histone H2B and the enhanced yellow fluorescent protein (eYFP), allowing detection of infected B cells and plasma cells. Since the kinetics of the immune response has been shown to be altered in IL-21R^-/-^ mice [21,23], we performed a time course analysis of MHV68 infection. Although the overall kinetics of infection were similar in wild type (wt) and IL-21R^-/-^ mice, the magnitude was greatly reduced in IL-21R^-/-^ mice (Fig 1 and Table 1). At day 14 post-infection, the percentage of YFP^+^ splenocytes was higher in wt mice than in IL-21R^-/-^ mice (0.053% and 0.013% respectively), but the difference was not statistically significant. Although the frequency of YFP^+^ cells increased in both strains after d14, the increase was significantly higher in wt mice. The percentage of YFP^+^ splenocytes in wt mice increased to 0.378% at day 16 (ca. 7-fold increase) and peaked at 0.426% at day 18 post-infection (ca. 8-fold increase) before falling to 0.174% at day 20. In IL-21R^-/-^ mice, the percentage of YFP^+^ cells increased to only 0.026% at d16 (ca. 2-fold increase) before peaking at 0.047% at day 18 (ca. 3.6-fold increase). Similar to wt mice, the YFP^+^ population was contracting at day 20 in IL-21R^-/-^ mice, with only 0.022% YFP^+^ cells.

**Table 1:** Average number of YFP^+^ cells per spleen.

	Total YFP+ cells (SEM)^a^		Total YFP+ GC B cells (SEM)^b^		Total YFP+ PCs (SEM)^c^	
	C57Bl6	Il21R^-/-^	C57Bl6	Il21R^-/-^	C57Bl6	Il21R^-/-^
d14	21,129 (6,898)	6,012 (1,256)	14,360 (4998)	2,515 (644)	4,034 (1,398)	1,127 (264)
d16	313,528 (117,143)	17,966 (3,414)	273,788 (110,482)	9,772 (1,929)	37,081 (13,599)	1,823 (327)
d18	254,735 (72,400)	20,579 (5,316)	200,146 (56,323)	14,089 (4170)	29,466 (7,862)	1,088 (248)
d20	84,718 (25,889)	9,637 (2,317)	65,518 (20,896)	4,648 (1,019)	7,641 (2,145)	3)

a. Calculated from mice infected in Fig 1A and 1B.

b. Calculated from mice infected in Fig 5A and 5B.

c. Calculated from mice infected in Fig 2C and 2D.

### IL-21 signaling is required for optimal generation of infected plasma cells

Because of the well-established role for IL-21 in generating humoral memory, we next analyzed the plasma cell populations in IL-21R^-/-^ mice. Analysis of the total plasma cell response from days 14–20 post-infection showed that there is a transient defect in the overall plasma cell response at day 16 post-infection, but that there was no significant difference in the overall differentiation of plasma cells at any other time points analyzed (Fig 2A and 2B). This is consistent with previous reports that showed that IL-21 is not required for generation of short-lived plasma cells [19,23], which most likely make up the majority of plasma cells present at the time points analyzed. Analysis of the MHV68 infected (YFP^+^) plasma cell population showed that, although there were significantly fewer total infected splenocytes in IL-21R^-/-^ mice (Fig 1), approximately 20% of infected splenocytes in both wt and IL-21R^-/-^ mice had a plasma cell phenotype at day 14 post-infection (Fig 2C and 2D). This percentage dropped to ca. 10% at day 16 post-infection in both strains of mice, and remained relatively steady in wt mice through day 20 post-infection. However, in IL-21R^-/-^ mice, the percentage of infected plasma cells continued to fall and constituted only 4.7% of the YFP^+^ population at day 20. Because there are no reliable markers to differentiate follicular derived from extra-follicular plasma cells, it is impossible to know the origin of infected plasma cells. However, since the defect in percentage of infected plasma cells is only apparent at the later time points, it is tempting to speculate that at days 18 and 20 post-infection, both extra-follicular and follicular-derived plasma cells contribute to the infected plasma cell pool in wt mice. Since IL-21R^-/-^ mice lack follicular derived plasma cells, the infected plasma cell fraction in these mice is likely made up of only extra-follicular-derived plasma cells, resulting in a reduced frequency as this population wanes over time.

**Fig 2:**
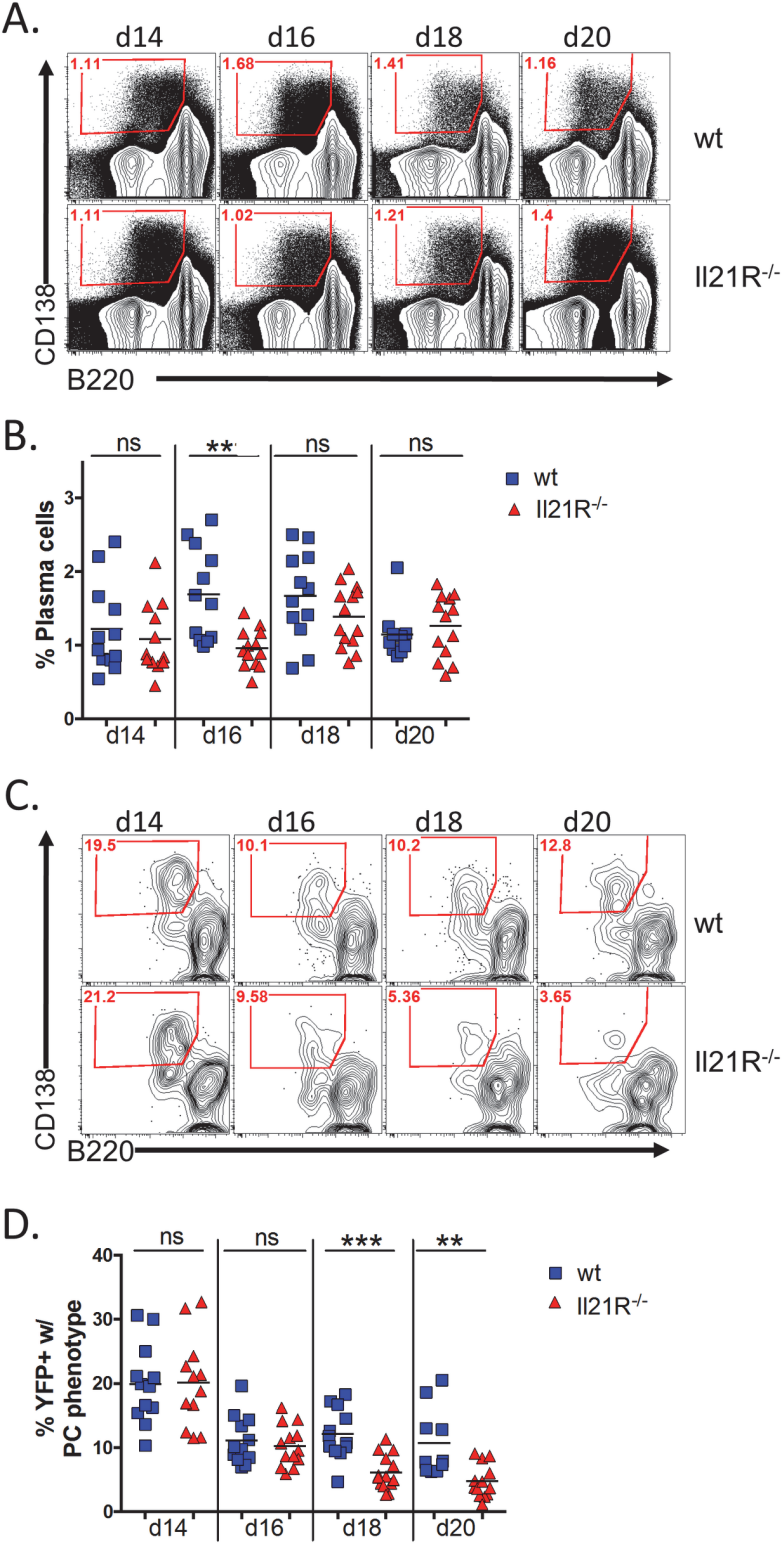
IL-21 is required for generation of infected PCs at late time points during the onset of latency. Mice were infected intranasally with 1,000 pfu of MHV68-H2bYFP and splenocytes were harvested at the indicated time points. (A) Representative flow plots showing the gating strategy to identify plasma cells. Plots were gated on CD3^-^, CD4^-^, CD8^-^ splenocytes. Plasma cells were defined as B220^neg-lo^, CD138^hi^ (red gated area). (B) Quantitation of the percentage of plasma cells present at the indicated time points. ns = not significant, **, p = 0.001. (C) Representative flow plots showing the percentage of infected cells that had a plasma cell phenotype (red gated area). Flow plots were gated on CD3^-^, CD4^-^, CD8^-^, YFP^+^ splenocytes and plasma cells were defined as in (A). (D) Quantitation of the percentage of YFP^+^ cells that had a plasma cell phenotype. ns = not significant; **, p = 0.0003; ***, p = 0.0025. Results in (B and D) were compiled from 3 independent experiments at each time point with 3–6 mice per group. Each symbol represents a single mouse and the horizontal lines represent the mean frequency.

**Fig 3:**
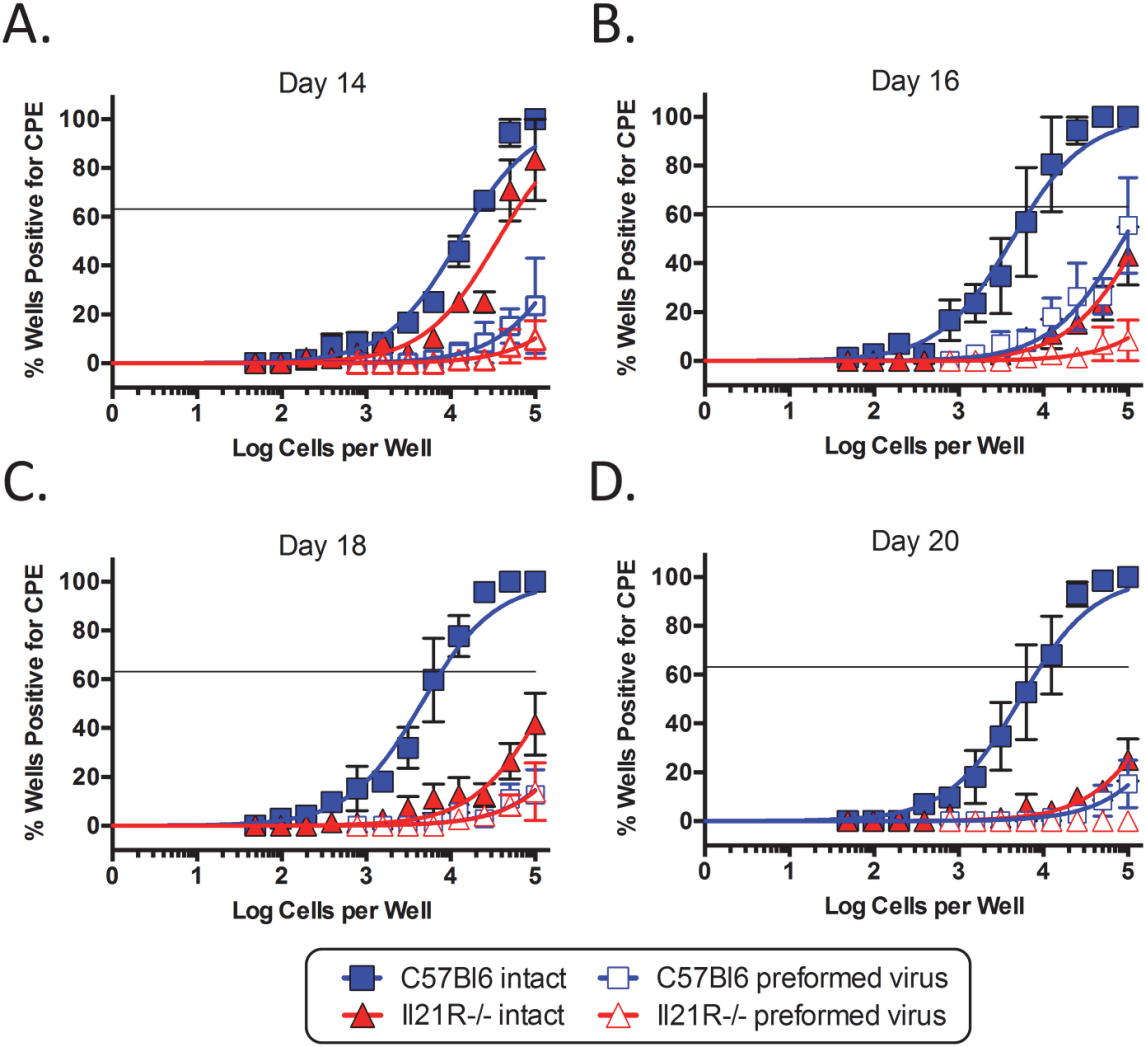
IL-21 signaling is required for efficient viral reactivation from splenocytes. To determine the frequency of splenocytes capable of reactivating virus, limiting dilution analysis was performed on splenocytes from mice infected intranasally with 1,000 pfu of MHV68-H2bYFP and harvested at days (A) 14, (B) 16, (C) 18 and (D) 20 post-infection. Serial dilutions of splenocytes were plated on MEFs and the percentage of wells positive for cytopathic effect (CPE) was determined 14 days post-plating. Mechanically disrupted splenocytes were plated in parallel to detect the presence of preformed infectious virus. Results are from 3 independent experiments at each time point with 3–6 mice per group.

Since splenic reactivation of MHV68 has been shown to occur predominantly from plasma cells [30], the defect in generating infected plasma cells suggested that splenocytes from IL-21R^-/-^ mice may reactivate less efficiently than those from wt mice. Because of this, we performed limiting dilution analyses to determine the frequency of splenocytes capable of reactivating virus. At day 14 post-infection, the frequency of splenocytes capable of reactivating virus was slightly lower in IL21R^-/-^ mice, with 1 in 60,885 reactivating cells compared to 1 in 21,515 in wt mice (Fig 3A and Table 2). At days 16 and 18 post-infection, whereas the frequency increased to ca. 1 in 7,500 cells in wt mice, it dropped dramatically in IL-21R^-/-^ mice to ca. 1 in 2.3x10^5^ cells (Fig 3B and 3C and Table 2). At day 20 post-infection, the frequency of reactivating cells from wt mice had contracted to 1 in 9,162 cells reactivating virus, whereas in IL21R^-/-^ mice the frequency had fallen further to 1 in 5.1x10^5^ cells (Fig 3D and Table 2). Since the overall frequency (Fig 1) and absolute number (Table 1) of infected cells in IL21R^-/-^ mice increased from days 14 to 18, but the frequency of splenocytes capable of reactivation decreased over this time, this indicates that the defect in reactivation was due not only to the reduced frequency of infected cells, but also to the reduced ability of infected cells to reactivate virus in IL-21R^-/-^ mice.

**Table 2:** Correlation between infected plasma cells and reactivating cells.

	Total YFP+ cells (Frequency^-1^)^a^		YFP+ Plasma cells (Frequency^-1^)^b^		Reactivating cells (Frequency^-1^)^c^	
	C57Bl6	Il21R^-/-^	C57Bl6	Il21R^-/-^	C57Bl6	Il21R^-/-^
d14	2,879	13,812	15,151	73,529	21,515	60,885
d16	314	6,369	3,576	64,102	7,393	234,970
d18	372	3,543	3,317	68,493	7,734	232,379
d20	915	8,347	9,881	172,413	9,162	514,149

a. Calculated from mice infected in Fig 1A and 1B.

b. Calculated from mice infected in Fig 2C and 2D.

c. Calculated from mice infected in Fig 3A–3D.

To determine if the defect in the ability of infected splenocytes to reactivate could be attributed to the reduced frequency of virus infected plasma cells, the frequency of YFP^+^ plasma cells was calculated and compared to the frequency of splenocytes that were capable of reactivation (Table 2). At day 14 post-infection, the frequency of YFP^+^ plasma cells correlated well with the frequency of reactivating cells in both strains, suggesting that the majority of infected plasma cells are capable of *ex vivo* reactivation during the early establishment of infection. At days 16 and 18 post-infection, the frequency of YFP^+^ plasma cells was approximately 2-fold higher than the frequency of reactivating cells in wt mice. This is consistent with our previous data showing that approximately 50% of infected plasma cells reactivate virus *ex vivo* at the peak of latency [30]. Although the frequency of reactivating cells dropped in IL-21R^-/-^ mice at days 16 and 18, the frequency of infected plasma cells increased, albeit slightly. However, the difference in the frequency of infected plasma cells and reactivating cells was only ca. 3.5-fold at these time points. At day 20 post-infection, there was good correlation between the frequencies of reactivating cells and YFP^+^ cells in wt mice, indicating that during the beginning of the contraction phase of splenic infection, the majority of infected plasma cells are capable of reactivating virus upon explant. In IL-21R^-/-^ mice, the difference in the frequency of infected plasma cells and the frequency of reactivating cells was 3-fold at day 20. Taken together, this data shows that the defect in reactivation in IL-21R^-/-^ mice correlates well with the reduced frequency of infected plasma cells. Additionally, the reduced ratio of YFP^+^ plasma cells to reactivating cells in IL-21R^-/-^ mice suggests that infected plasma cells in IL-21R^-/-^ mice may be less efficient at reactivating virus. However, these differences are small, so although suggestive, we cannot conclusively say that infected plasma cells from IL-21R^-/-^ mice are less efficient at reactivating virus.

### Reduced MHV68 infection of germinal center B cells in IL-21R^-/-^ mice

**Fig 4:**
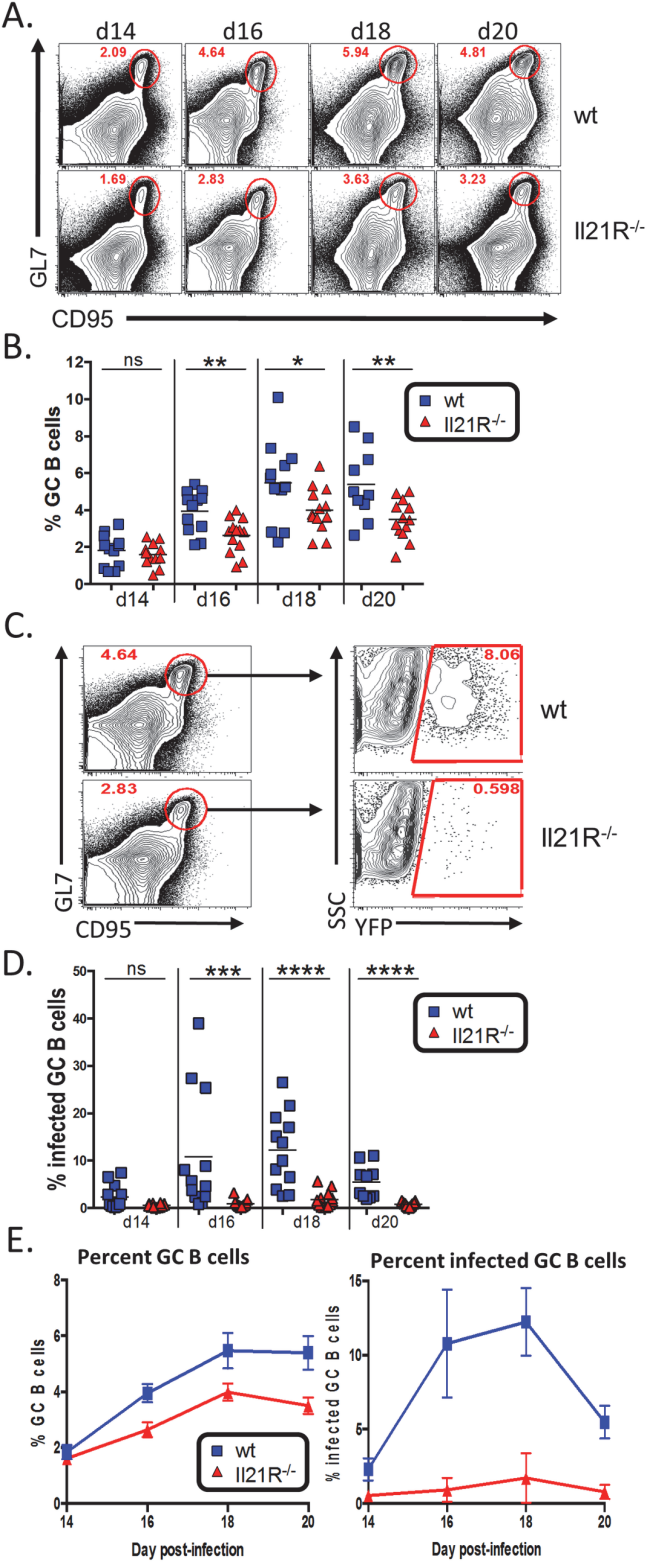
Reduced germinal center response in IL-21R^-/-^ mice. Mice were infected intranasally with 1,000 pfu of MHV68-H2bYFP and splenocytes were harvested at the indicated time points. (A) Representative flow plots showing reduced germinal center B cells in IL-21R^-/-^ mice. Flow plots were gated on CD3^-^, CD4^-^, CD8^-^, B220^+^, and germinal center B cells were defined as CD95^hi^ GL7^hi^. (B) Quantitation of the percentage of B cells that have a germinal center phenotype at the indicated time points. d14, ns = not significant; d16, p = 0.0052; d18, p = 0.0462; d20, p = 0.0178. (C) Gating strategy to determine the percentage of germinal center B cells that are infected based on YFP expression. Germinal center B cells are defined as in (A). (D) Quantitation of the percentage of germinal center B cells that are infected at the indicated time points. d14, ns = not significant; d16, p = 0.0001, d18 and d20, p< 0.0001. (E) Comparison of the increase in total germinal center B cells (left graph) versus the increase in the percentage of infected germinal center B cells (right graph). Results in B, D and E are from 3 independent experiments at each time point with 3–6 mice per group. Error bars represent SEM.

Since IL-21 signaling has been shown to play a role in maintenance of the germinal center response [20,23], we next analyzed the germinal center B cell population in infected IL-21R^-/-^ mice. Similar to the overall kinetics of MHV68 infection (Fig 1), the kinetics of the germinal center response were similar in wt and IL-21R^-/-^ mice, peaking at day 18 post-infection (Fig 4A and 4B). However, the overall magnitude of the response was significantly lower in IL-21R^-/-^ mice. At day 14 post-infection, there was no significant difference in the percentage of B cells that exhibited a germinal center phenotype (Fig 4B). By day 16 post-infection, the percentage of germinal center B cells in wt mice had risen to a significantly higher level than in IL-21R^-/-^ mice, and remained significantly higher through day 20.

Although there was a statistically significant decrease in the overall GC population in IL-21R^-/-^ mice (<2-fold) (Fig 4A and 4B), it was not as severe as the defect in overall MHV68 infection (~10-fold at days 16–20 post-infection) (Fig 1A and 1B). This is notable because it has previously been shown that the frequency of MHV68 infection in B cells in immunocompetent mice directly correlates with the magnitude of the germinal center response [9,36] as well as the level of infection in germinal center B cells [5]. This disparity between overall infection and the germinal center response in IL-21R^-/-^ mice was reflected in a significant reduction in the percentage germinal center B cells that were infected in IL-21R^-/-^ mice (Fig 4C and 4D). In wt mice, the mean percentage of infected germinal center B cells was ca. 10% at days 16 and 18 post-infection. However, in IL-21R^-/-^ mice, infected cells made up less than 1% of the germinal center population at d16 and peaked at 1.7% at d18. To further illustrate this, Fig 4E shows a comparison of the percentage of germinal center B cells over time (left graph) versus the percentage of infected germinal center B cells over time (right graph). IL-21R^-/-^ mice are capable of generating a significant, albeit reduced, population of germinal center B cells that expands over time similar to wt mice, with less than a 1.5 fold difference at all time points. However, the difference in the percentage of infected germinal center B cells is much greater between wt and IL-21R^-/-^ mice (ca. 10 fold). Taken together, this data indicates that generation of MHV68 infected germinal center B cells is much more reliant of IL-21 signaling than uninfected B cells.

**Fig 5:**
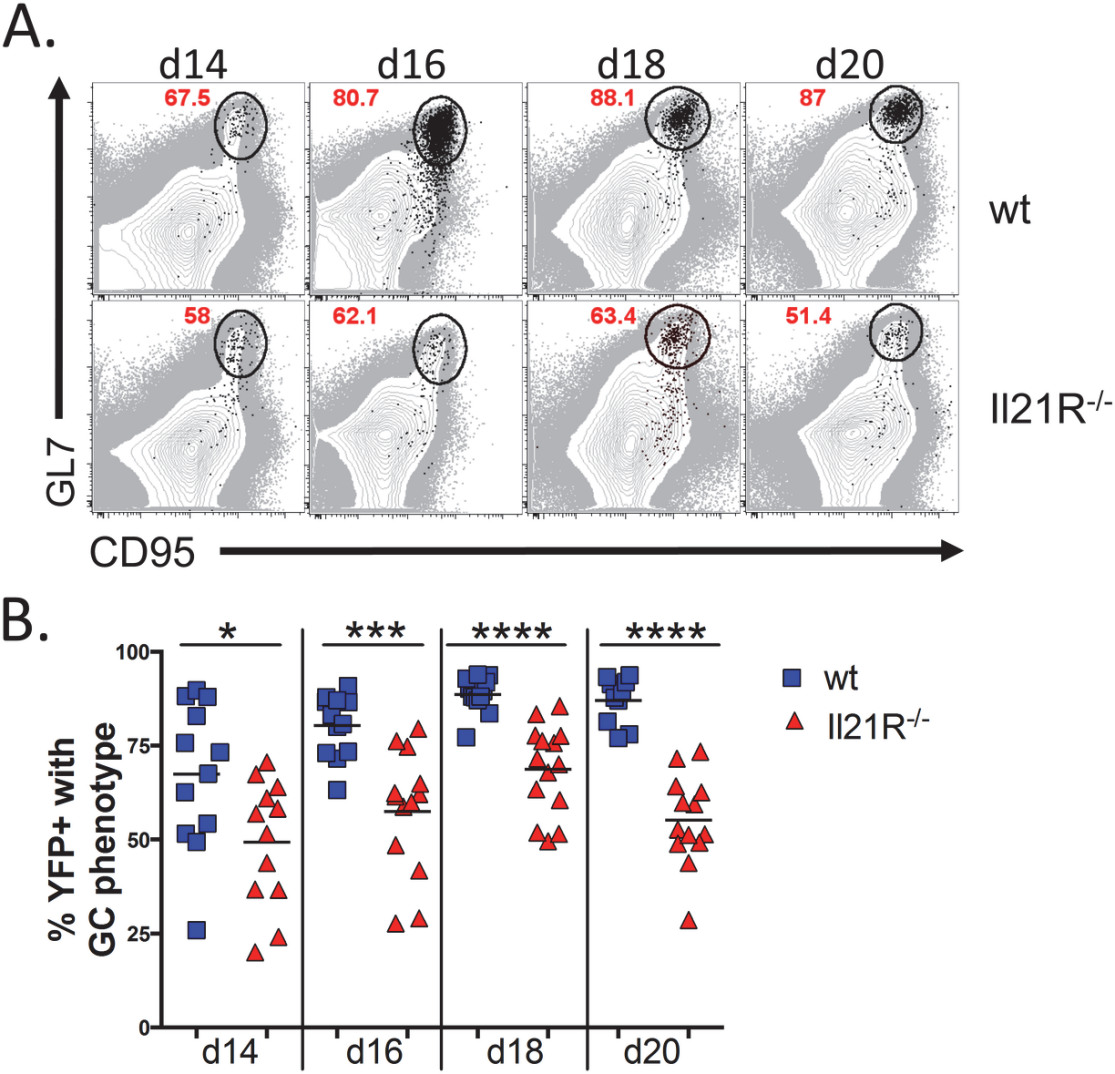
Reduced frequency of infected B cells with a germinal center phenotype in IL21-R^-/-^ mice. Mice were intranasally infected with 1,000 pfu of MHV68-H2bYFP and splenocytes were harvested at the indicated time points. (A) Representative flow plots of YFP^+^ germinal center B cells (black dots) overlaid onto the total B cell population (gray), showing that a reduced percentage of YFP^+^ B cells have a germinal center phenotype in IL-21R^-/-^ mice. Flow plots were gated on CD3^-^, CD4^-^, CD8^-^, B220^+^, YFP^+^, and germinal center B cells were defined as CD95^hi^ GL7^hi^. (B) Quantitation of the percentage of YFP^+^ B cells that have a germinal center phenotype. Each symbol represents a single mouse. Results are from 3 independent experiments at each time point with 3–6 mice per group. d14, p = 0.0252; d16, p = 0.0001; d18 and d20, p<0.0001.

In accordance with the reduced frequency of germinal center B cells that were infected, analysis of the total MHV68 infected B cell populations showed that a significantly lower percentage of the infected B cells in IL21R^-/-^ mice had differentiated into germinal center B cells at all time points examined (Fig 5A and 5B). In wt mice, nearly 90% of the infected B cells exhibited a germinal center phenotype at the peak of infection (day 18), whereas less than 70% of infected B cells in IL21R^-/-^ mice had a germinal center phenotype at the peak of infection. Although there was a significant fraction of non-germinal center infected B cells in IL-21R^-/-^ mice, these cells still expressed high levels of CD95 and had intermediate levels of GL7, indicating that they were activated, but less efficient at fully differentiating into germinal center B cells.

**Fig 6:**
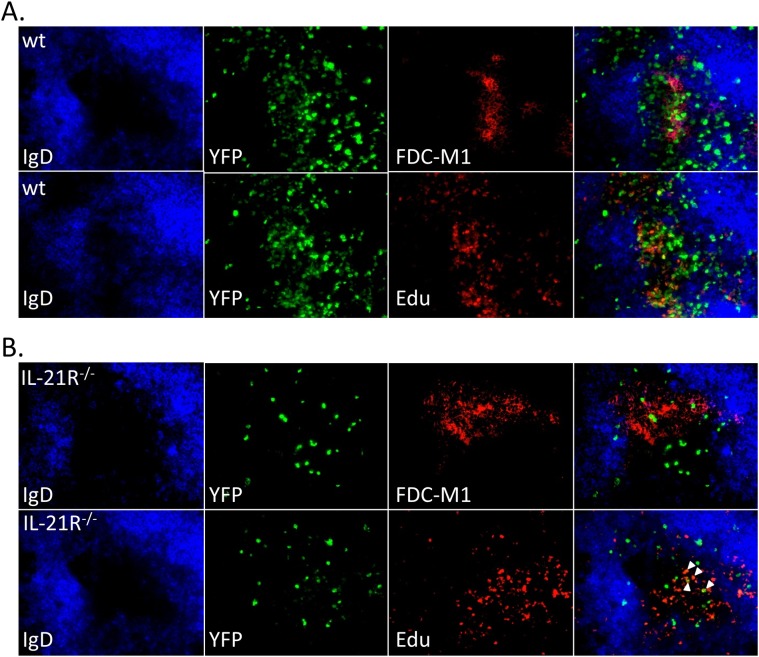
Reduced infection in germinal centers in IL-21R^-/-^ of mice. Representative spleen sections from mice infected intranasally with 1,000 pfu of MHV68-H2bYFP and harvested at day 16 post-infection. Mice were injected intraperitoneally with Edu 5 hours before spleens were harvested to detect proliferating centroblasts in the dark zone of germinal centers. Sections were co-stained with anti-IgD to delineate the mantle zone surrounding germinal centers and anti-FDC-M1 to detect the follicular dendritic cell network in light zone in sections from (A) wt mice and (B) IL-21R^-/-^ mice. Arrows indicate Edu^+^ YFP^+^ cells in IL-21R^-/-^ mice.

To determine if infected cells localized to germinal centers in IL-21R^-/-^ mice, spleen sections were stained for markers to detect the dark and light zones of germinal centers. To detect proliferating centroblasts in the dark zone, mice were injected intraperitoneally with Edu 5 hours before spleens were harvested. Sections were co-stained with anti-IgD to delineate B cell follicles and anti-FDC-M1 to delineate the follicular dendritic cell network located in the light zone of germinal centers. In wt mice, germinal centers with numerous YFP^+^ cells were easily detectable (Fig 6A). These germinal centers contained numerous proliferating YFP^+^ cells that localized predominantly to the dark zone, as well as non-proliferating YFP^+^ cells found predominantly in the light zone. In IL-21R^-/-^ mice there were far fewer germinal centers containing YFP^+^ cells, with less than 1 infected germinal center per section. Germinal centers in IL-21R^-/-^ mice appeared to be relatively normal with distinct dark and light zones (Fig 6B). However, there were fewer YFP^+^ cells per germinal center and only a few these infected cells were proliferating, consistent with the reduced frequency of infected germinal center B cells (Fig 4C and 4D).

### Germinal center B cell populations are skewed toward a centrocyte phenotype in IL-21R^-/-^ mice

**Fig 7:**
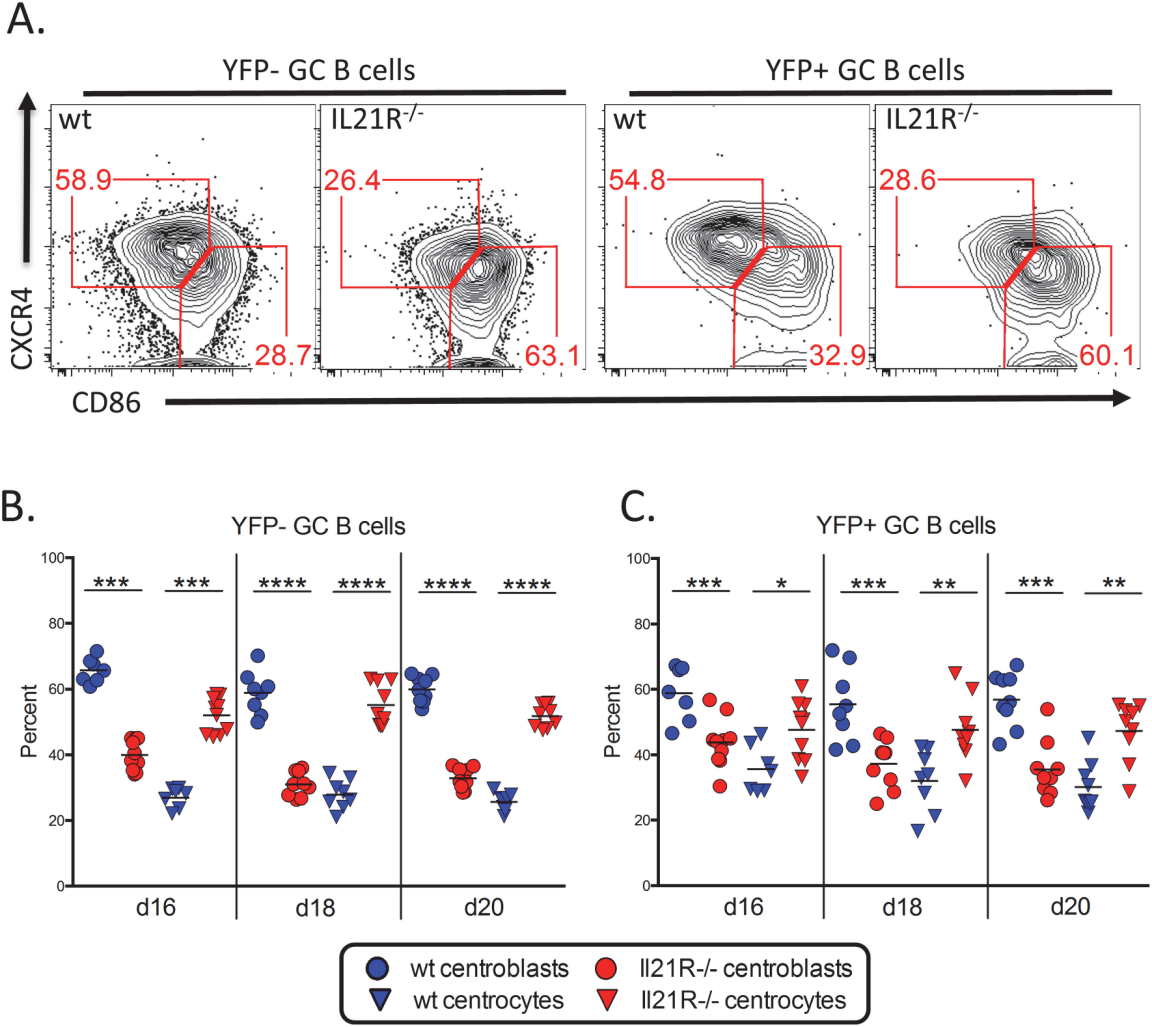
Altered GC B cell populations in IL-21R-/- mice. Mice were infected intranasally with 1,000 pfu of MHV68-H2bYFP and splenocytes were harvested at the indicated time points. (A) Representative flow plots showing germinal center B cells populations. Plots are gated on YFP^-^ or YFP^+^ germinal center B cells (CD3^-^, CD4^-^, CD8^-^, B220^+^, CD95^hi^, GL7^hi^). Centroblasts are defined as CXCR4^hi^, CD86^lo-neg^ and centrocytes are defined as CXCR4^lo-neg^, CD86^hi^. Quantitation of (B) uninfected and (C) infected centroblast and centrocyte populations. *, p<0.05; **, p<0.01; ***, p<0.001; ****,p<0.0001.

We have previously shown that MHV68 infected germinal center B cells have a similar proportion of centroblasts and centrocytes as germinal center B cells induced during T-dependent immune responses [5,37]. To see if the loss of IL21 signaling had any impact on these populations, we quantitated the frequency of each cell type (Fig 7A). At all time points examined, there was a significantly lower percentage of centroblasts and a corresponding increase in the percentage of centrocytes in both the uninfected and infected germinal center B cell populations of IL21R^-/-^ mice compared to wt mice (Fig 7B and 7C). This suggests that germinal center B cells may become arrested at the centrocyte stage in IL-21R^-/-^ mice, and that IL-21 produced by T_FH_ cells is required for re-entry to the dark zone for additional rounds of proliferation and somatic hypermutation. This skewing of the germinal center B cell populations towards the non-proliferating centrocyte phenotype is consistent with previous work showing that the overall population of germinal center B cells is less proliferative and has reduced somatic hypermutation in the absence of IL-21 signaling [21]. This also suggests that the reduced proliferation of YFP^+^ germinal center B cells in the absence of IL-21 signaling may play a role in the reduced frequency of YFP^+^ cells (Fig 1A and 1B) as well as the frequency of YFP^+^ cells with a germinal center phenotype (Fig 4C and 4D).

### The requirement for IL-21 signaling in establishment of viral latency is intrinsic to B cells

Since T_FH_ cells also express IL-21R and respond to IL-21 signaling, any effects on infected B cells may be due to the inability of IL-21R^-/-^ T_FH_ cells to provide sufficient help during the germinal center reaction. It has previously been shown that the defects in the germinal center response and in generating humoral memory in IL-21R^-/-^ mice are intrinsic to B cells [19–21]. However, it is possible that although the T_FH_ cells generated in IL-21R^-/-^ mice can drive a substantial germinal center reaction, they may lack certain functions that, while not absolutely critical for the GC reaction, are critical for viral infection. To address this issue, we generated mixed IL-21R^+/+^/IL-21R^-/-^ bone marrow chimeric mice. In this setting, both IL-21R deficient and IL-21R sufficient B cells will be in direct competition for IL-21R sufficient CD4 T cell help. IL21R^+/+^ mice expressing the Ly5.1 congenic marker were lethally irradiated and reconstituted with a 50:50 mixture of bone marrow from IL-21R^+/+^/Ly5.1^+^ and IL-21R^-/-^/Ly5.2^+^ mice. Eight weeks after reconstitution, mice were infected intranasally with 1,000 pfu of MHV68-H2bYFP and spleens were harvested at days 14 and 18 post-infection.

**Fig 8:**
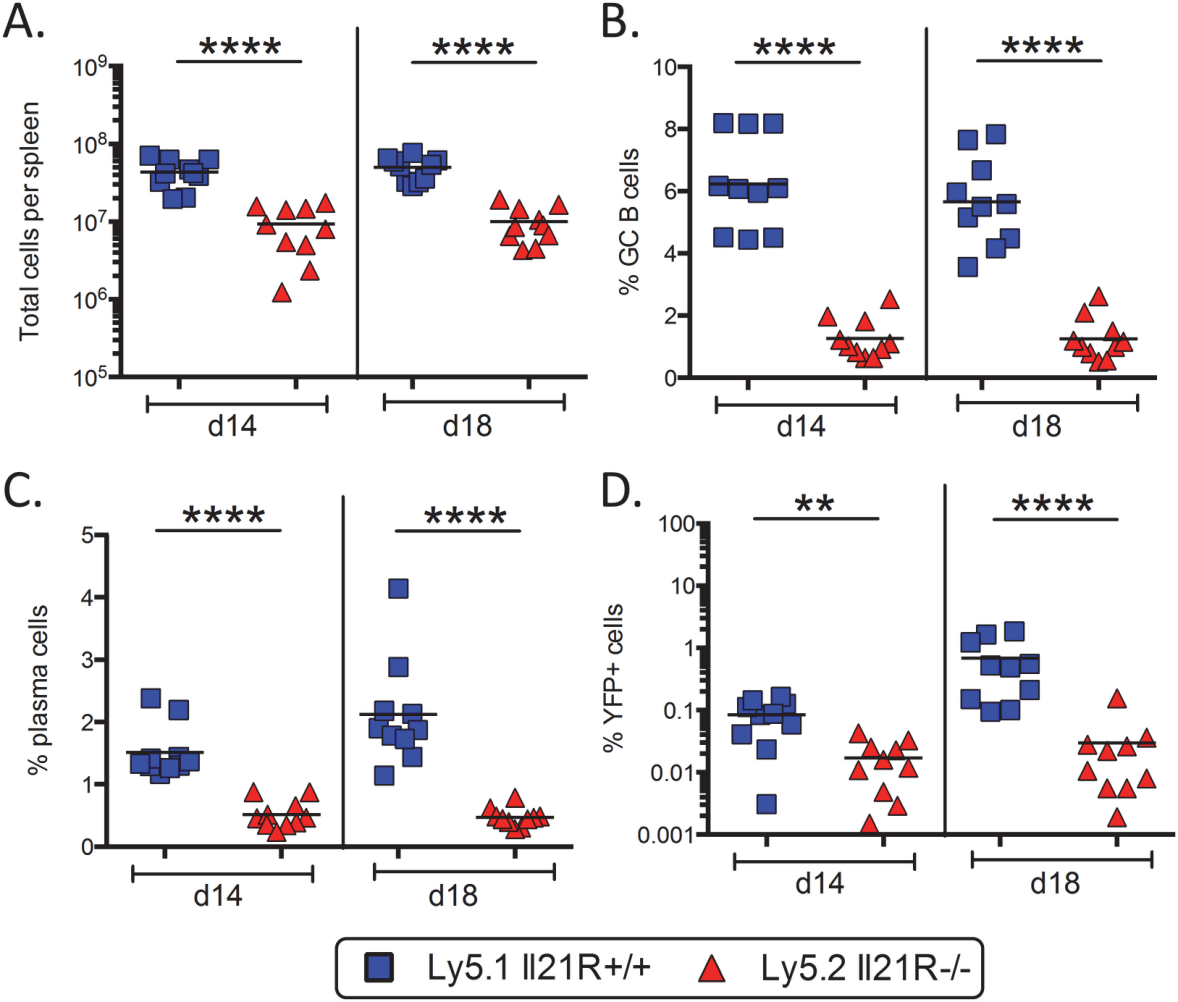
Requirement for IL-21 signaling in establishment of MHV68 infection is intrinsic to B cells. IL21R^+/+^/IL21R^-/-^ mixed bone marrow chimeric mice were infected intranasally with 1,000 pfu of MHV68-H2bYFP and spleens were harvested at the indicated days. (A) Total number of Ly5.1^+^ IL21R^+/+^ and Ly5.2^+^ Il21R^-/-^ splenocytes harvested. (B) Percentage of B cells with a germinal center phenotype. Cells were gated on Ly5.1^+^ or Ly5.2^+^, B220^+^ CD3^-^, CD4^-^, CD8^-^ and germinal center B cells were defined as CD95^hi^ GL7^hi^. (C) Percentage of splenocytes with a plasma cell phenotype. Cells were gated on CD3^-^, CD4^-^, CD8^-^ and plasma cells were defined as B220^neg-lo^ CD138^hi^. (D) Percentage of splenocytes that were MHV68 infected (YFP+). Cells were gated on CD3^-^, CD4^-^, CD8^-^ splenocytes. **, p = 0.0029; ****,p<0.0001.

As shown in Fig 8A, although there were robust populations of both Ly5.1^+^ and Ly5.2^+^ splenocytes, there were significantly more Ly5.1^+^ IL-21R^+/+^ splenocytes present. Additionally, the percentage of B cells that had a germinal center phenotype was significantly higher in the Ly5.1^+^ IL-21R^+/+^ B cell fraction than in Ly5.2^+^ IL-21R^-/-^ B cells (Fig 8B). Notably, the defect in the germinal center response in Ly5.2^+^ IL-21R^-/-^ B cells compared to Ly5.1^+^ IL-21R^+/+^ B cells was much greater than the defect in IL-21R^-/-^ mice compared to wt mice, where there was less than a 2-fold defect at each time point analyzed (Fig 3B). The percentage of plasma cells was also significantly higher in the Ly5.1^+^ IL-21R^+/+^ fraction (Fig 8C), although there was no defect in generating plasma cells in IL-21R^-/-^ with the exception of day 16 (Fig 2B). Importantly, MHV68 infection was significantly higher in Ly5.1^+^ IL-21R^+/+^ B cells (Fig 8D). This indicates that the defect in IL-21 signaling that results in reduced viral infection is intrinsic to B cells and not due to a role for IL-21 signaling in CD4 T cells.

## Discussion

Passage through the germinal center reaction is an important step in the infection cycle of gammaherpesviruses, and is thought to provide a mechanism for infected cells to gain access to the memory B cell pool. We have previously shown that MHV68 requires signals from T_FH_ cells during the germinal center reaction for efficient establishment of infection during the onset of latency [9]. We extended this finding here by identification of IL-21, a T_FH_ cell derived cytokine, as essential for efficient establishment of MHV68 infection. The lack of IL-21 signaling in B cells impacts MHV68 infection at multiple levels, resulting in significantly reduced infection.

In addition to a significant reduction in the frequency of infected cells in IL-21R^-/-^ mice, there are also significantly fewer infected B cells from IL-21R^-/-^ mice that have a germinal center phenotype. Furthermore, those infected B cells that do gain access to the germinal center pool are skewed towards a centrocyte phenotype. This skewing towards the non-proliferating fraction of germinal center B cells is likely to be a major contributing factor to the reduced level of infection. This accumulation in the centrocyte fraction in IL-21R^-/-^ mice suggests that IL-21 signaling is required for differentiation of centrocytes to centroblasts and re-entry into the dark zone of the germinal center for additional rounds of proliferation and somatic hypermutation. Previous work showing that germinal center B cells in mice deficient in IL-21 signaling are less proliferative and have fewer mutations in their immunoglobulin genes supports this hypothesis [21]. Additionally, the inability to exit the centrocyte stage may explain why mice defective in IL-21 signaling are unable to generate humoral memory.

It has previously been shown that the level of infection in B cells is proportional to the magnitude of the germinal center response [9,36], so it is somewhat surprising that the defect in infection was much greater than the defect in generating germinal center B cells in IL-21R^-/-^ mice. A possible explanation for this could be that the germinal center response in IL-21R^-/-^ mice is being sustained by an influx of newly activated B cells into existing germinal centers. Intra-vital imaging studies have shown that germinal centers are not closed structures, and that B cells can participate in the germinal center reaction by entering existing germinal centers [38,39]. Since IL-21R^-/-^ B cells do not undergo somatic hypermutation, they are not able to out-compete these newly activated B cells for T_FH_ cell help, resulting in a constant influx of newly activated B cells into the germinal center. Because the pool of latently infected B cells is limited, there is unlikely to be a continuing influx of newly activated infected B cells into germinal centers—particularly given the observed defect in virus reactivation in IL21R-/- mice which may serve as an important source of virus for establishment of MHV68 B cell latency (see Fig 3). In support of this hypothesis, when IL-21R^-/-^ B cells were put in direct competition with IL-21R^+/+^ B cells in mixed bone marrow chimeras, there was a much more dramatic defect in their ability to differentiate into germinal center B cells compared to the difference in the germinal center response in IL-21R^-/-^ mice compared to wt mice (see Fig 8B).

Although the defect in reactivation in IL-21R^-/-^ mice is linked to the reduced frequency of infected plasma cells, what impact this has on MHV68 infection is not clear. There is a significant reduction in the frequency of splenocytes capable of reactivation at later time points, but the defect is minor at day 14 post-infection, suggesting that IL-21 signaling is not required for virus production during initial seeding of the spleen. However, the reduced frequency of infected plasma cells at later time points suggests that the defect is in generating follicular-derived plasma cells, consistent with the known role for IL-21. The lack of infected follicular-derived plasma cells may impact the level of infection by reducing the level of *de novo* infection. However, we currently do not know if infected plasma cells are producing virus *in vivo* at these later time points. Although infected plasma cells reactivate virus *ex vivo*, it is less clear whether they are the source of the low levels of preformed infectious virus detected in disrupted splenocytes explanted from wt mice at days 18 and 20 post-infection.

Another factor that may be contributing to reduced infection in IL-21R^-/-^ mice is altered viral gene expression. Viral genes essential for creating a cellular environment that is conducive to gammaherpesvirus latency may be dependent on IL-21 signaling for expression. In human B cells infected with EBV, IL-21 has been shown to repress the viral transcriptional activators Zta and Rta during the first few days in culture [40]. In latently infected EBV transformed cell lines, IL-21 up-regulates expression of the CD40L mimic LMP-1 in type I latency cell lines [41–43], whereas it down-regulates expression of EBNA-2 [41] and LMP2a [42] in type III latency cell lines. Identification of MHV68 encoded genes that are regulated by IL-21 will be important in elucidating the mechanism of how IL-21 signaling supports establishment of infection.

A longstanding question about MHV68 infection concerns what type of B lineage cell the virus infects. Although MHV68 infection can be found in naïve B cells, germinal center B cells and plasma cells, it remains unclear whether or not the virus infects naïve B cells that subsequently undergo differentiation to germinal center B cells and plasma cells, or if the virus infects each cell type directly. We have previously shown that the ability MHV68 to establish infection is severely impacted in the absence of a robust germinal center response [9], and it can be argued that this is due to the lack of germinal center B cells available for infection. However the data presented here supports a model where MHV68 infects naïve B cells that undergo subsequent differentiation. During initial seeding of the spleen at day 14 in IL-21R^-/-^ mice, there is no significant reduction in the germinal center response and less than a 3 fold reduction in the frequency of splenocytes capable of reactivating virus, indicating that there is an abundant pool of target cells for infection as well as cells capable of producing virus. However, there is a significant reduction in the percentage of YFP^+^ B cells that have a germinal center phenotype in IL-21R^-/-^ mice, arguing against the direct infection model.

In conclusion, our data indicates that the defect in establishment of latency in the absence of IL-21 signaling occurs at the centrocyte stage of infection. In the absence of IL-21, centrocytes fail to transition back to centroblasts for additional rounds of proliferation and fail to exit the germinal center reaction as either memory B cells or long lived plasma cells. The failure to proliferate and to differentiate into plasma cells contribute to reduced infection, and further serves to illustrate how gammaherpesviruses have evolved to take advantage of normal host differentiation pathways to establish infection.

## Materials and Methods

### Ethics statement

This study was carried out in strict accordance with the recommendations in the Guide for the Care and Use of Laboratory Animals of the National Institutes of Health. The protocol was approved by the Emory University Institutional Animal Care and Use Committee, and in accordance with established guidelines and policies at Emory University School of Medicine (Protocol Number: YER-2002245-031416GN).

### Mice, virus and infections

C57Bl/6J mice were purchased from Jackson laboratories (ME). IL-21R^-/-^ mice backcrossed onto the C57Bl/6 background were obtained from Warren Leonard and have been previously described [44]. Mice were housed within the Emory University Division of Animal Resources, and monitored quarterly via a sentinel program for the presence of Mycoplasma pulmonis, Mouse Hepatitis Virus, Sendai virus, MPV-1, MPV-2, NS-1 (Murine Parvoviruses), TMEV (Theiler’s Murine Encephalomyelitis Virus), PVM (Pneumonia Virus of Mice), MVM (Minute Virus of mice), MNV (Mouse Norovirus), Mouse Rotavirus (EDIM), Reovirus-3 and examinations to rule-out ectoparasites and helminths. In addition, they are assessed annually for LCMV (Lymphocytic Choriomeningitis Virus), K virus, Polyoma virus, and Mouse Adenovirus. Currently, Emory Division of Animal Resources managed mouse colonies have high level epizootic murine norovirus (MNV) and low level enzootic mouse parvovirus (MPV) infections.

All experiments were performed using female mice that were at least 8 weeks old, with 3 to 6 mice per group. All infections were performed using MHV68-H2bYFP, a transgenic virus that expresses a fusion protein consisting of histone H2B and eYFP and has been previously described [5]. For each experiment, C57Bl/6J mice were infected in parallel with 1,000 pfu of wild type MHV68 intranasally to differentiate between auto-fluorescing cells and YFP+ cells (described below under Flow cytometry). Prior to infection, mice were anesthetized with isofluorane and inoculated intranasally with 1,000 pfu of virus diluted in 20 ul of DMEM. At the indicated time points, mice were sacrificed by CO_2_ asphyxiation and spleens were harvested. To obtain single cell suspensions of splenocytes, spleens were homogenized and filtered through 100 um pore size nylon cell strainers (Becton-Dickinson, Franklin Lakes, NJ). Red blood cells were lysed with red blood cell lysis buffer (Sigma).

### Limiting dilution *ex vivo* reactivation

Limiting dilution analysis to determine the frequency of splenocytes capable of reactivating virus was performed as previously described [45,46]. Briefly, equivalent numbers of bulk splenocytes from either wt or IL-21R^-/-^ mice were pooled for each experiment, with 3–6 mice per group. Two fold serial dilutions, starting with 1x10^5^ splenocytes at the highest dilution, were plated out on MEF monolayers, with 24 replicates per dilution. The percentage of wells at each dilution that displayed CPE were scored 14 days after plating. To determine how much preformed infectious virus was present, splenocytes were resuspended in hypotonic medium consisting of 1/3X Dulbecco’s modified Eagle’s medium in the presence of 0.5 mm zirconia/silica beads (Biospec Products) and disrupted using a Minibeadbeater 16 (Biospec Products). This process kills 99% of cells, allowing pre-formed infectious virus to be distinguished from virus reactivating from latently infected cells.

### Flow cytometry

Single cell suspensions of splenocytes were resuspended in PBS containing 2% fetal bovine serum and stained on ice in the dark for 20 minutes. Antibodies used were PE conjugated anti-CD138, anti-CD86 and anti-Ly5.2 (BD Biosciences), PerCP conjugated anti-CD3, anti-CD4 and anti-CD8 (BD Biosciences) and anti-GL7 (eBioscience), PE-Cy7 conjugated anti-CD95 and anti-Ly5.1 (BD Biosciences), Pacific Blue conjugated anti-B220 (Biolegend), eFluor 660 conjugated anti-GL7 (eBioscience), and APC conjugated anti-CXCR4 and anti-CD138 (BD Biosciences), APC-Cy7 conjugated anti-CD8 (Biolegend) and APC-Cy7 conjugated anti-Ly5.1 (BD Biosciences). For each infection, C57Bl6/J mice were infected in parallel with wt MHV68. Gates for the YFP+ populations were set based on the level of auto-fluorescence detected in the FITC channel from mice infected with wt virus as shown in Fig 1A. Data was collected on an LSRII flow cytometer (BD Biosciences) and analyzed using FlowJo software (Tree Star Inc., http://www.flowjo.com).

### Tissue section preparation and immunofluoresence microscopy

Spleens were embedded in OCT media (Sakura Finetek) and flash frozen in liquid nitrogen chilled isopentane. Sections were cut at a thickness of 5 um and mounted on glass slides. Sections were re-hydrated at room temperature in PBS for 10 minutes and then blocked at room temperature with PBS containing 3% BSA for 20 minutes. FITC conjugated anti-GFP (Rockland Immunochemicals) was used to enhance the signal from YFP in infected cells. Cellular markers were detected with Alexa 647conjugated anti-IgD (BD Biosciences) and purified anti-FDC-M1 (BD Biosciences) followed by Alexa 546 conjugated anti-rat IgG (Invitrogen). All images were collected on an Axiovert 200M fluorescent microscope (Carl Zeiss) using Axiovision version 4.7 imaging software.

### Edu labeling and detection

To detect proliferating cells, mice injected intra-peritoneally with 100 mg of 5’-ethynyl 2’-deoxyuridine (EdU) (Invitrogen) 5 hours before spleens were harvested and frozen as described above. EdU detection was performed as previously described with a Click-it EdU Alexa Fluor 555 Imaging kit (C10338) according to the manufacturer’s protocol with the following modification. Unfixed sections were stained with fluorophore conjugated antibodies as described above, then fixed with 4% paraformaldehyde for 10 minutes at room temperature followed by permeabilization with 0.2% Triton-X in PBS for 10 minutes. EdU detection was then performed according to the manufacturer’s protocol.[5,9].

### Mixed bone marrow chimeras

B6.SJL-Ptprc^a^Pep3^b^/BoyJ (Ly5.1) mice (catalog no. 002014; The Jackson Laboratory) were lethally irradiated with 2 doses of 475 rads (950 total) at a 16 hour interval and reconstituted with a 50:50 mix of bone marrow from B6.SJL-Ptprc^a^Pep3^b^/BoyJ (Ly5.1) and IL-21R^-/-^ (Ly5.2) mice. A total of 1x10^7^ cells in 200 ul PBS were administered by tail vein injection. Mice were then rested for 8 weeks to allow for reconstitution, which was confirmed by flow cytometry of peripheral blood. Chimeric mice received polymyxin B and neomycin (Sigma-Aldrich) in acidified drinking water until reconstitution was confirmed.

### Statistical analysis

All data were analyzed using GraphPad Prism software (GraphPad Software, http://www.graphpad.com). Due to the asynchronous nature of splenic infection after intranasal inoculation, groups did not always have a Gaussian distribution as determined by the D’Agostino-Pearson omnibus normality test. Because of this, statistical significance was determined by the Mann-Whitney U test with a confidence level of 95%. The frequency of splenocytes capable of reactivating virus was determined by non-linear regression analysis. Frequencies were obtained from non-linear regression fit where the regression line intersected 63.2%, corresponding to the frequency at which one event is predicted to be present in a population.

## References

[1] Thorley-Lawson DA (2001) Epstein-Barr virus: exploiting the immune system. Nat Rev Immunol 1: 75–82. 11905817 10.1038/35095584

[2] Uchida J, Yasui T, Takaoka-Shichijo Y, Muraoka M, Kulwichit W, et al (1999) Mimicry of CD40 signals by Epstein-Barr virus LMP1 in B lymphocyte responses. Science 286: 300–303. 10514374 10.1126/science.286.5438.300

[3] Caldwell RG, Wilson JB, Anderson SJ, Longnecker R (1998) Epstein-Barr virus LMP2A drives B cell development and survival in the absence of normal B cell receptor signals. Immunity 9: 405–411. 9768760 10.1016/s1074-7613(00)80623-8

[4] Collins CM, Boss JM, Speck SH (2009) Identification of infected B-cell populations by using a recombinant murine gammaherpesvirus 68 expressing a fluorescent protein. J Virol 83: 6484–6493. 10.1128/JVI.00297-09 19386718 PMC2698576

[5] Collins CM, Speck SH (2012) Tracking murine gammaherpesvirus 68 infection of germinal center B cells in vivo. PLoS One 7: e33230 10.1371/journal.pone.0033230 22427999 PMC3302828

[6] Flano E, Kim IJ, Woodland DL, Blackman MA (2002) Gamma-herpesvirus latency is preferentially maintained in splenic germinal center and memory B cells. J Exp Med 196: 1363–1372. 12438427 10.1084/jem.20020890 PMC2193987

[7] Marques S, Efstathiou S, Smith KG, Haury M, Simas JP (2003) Selective gene expression of latent murine gammaherpesvirus 68 in B lymphocytes. J Virol 77: 7308–7318. 12805429 10.1128/JVI.77.13.7308-7318.2003 PMC164786

[8] Willer DO, Speck SH (2003) Long-term latent murine Gammaherpesvirus 68 infection is preferentially found within the surface immunoglobulin D-negative subset of splenic B cells in vivo. J Virol 77: 8310–8321. 12857900 10.1128/JVI.77.15.8310-8321.2003 PMC165249

[9] Collins CM, Speck SH (2014) Expansion of murine gammaherpesvirus latently infected B cells requires T follicular help. PLoS Pathog 10: e1004106 10.1371/journal.ppat.1004106 24789087 PMC4006913

[10] Takemori T, Kaji T, Takahashi Y, Shimoda M, Rajewsky K (2014) Generation of memory B cells inside and outside germinal centers. Eur J Immunol 44: 1258–1264. 10.1002/eji.201343716 24610726

[11] Oracki SA, Walker JA, Hibbs ML, Corcoran LM, Tarlinton DM (2010) Plasma cell development and survival. Immunol Rev 237: 140–159. 10.1111/j.1600-065X.2010.00940.x 20727034

[12] Victora GD, Nussenzweig MC (2012) Germinal centers. Annu Rev Immunol 30: 429–457. 10.1146/annurev-immunol-020711-075032 22224772

[13] Zotos D, Tarlinton DM (2012) Determining germinal centre B cell fate. Trends Immunol 33: 281–288. 10.1016/j.it.2012.04.003 22595532

[14] Gitlin AD, Shulman Z, Nussenzweig MC (2014) Clonal selection in the germinal centre by regulated proliferation and hypermutation. Nature 509: 637–640. 10.1038/nature13300 24805232 PMC4271732

[15] Chtanova T, Tangye SG, Newton R, Frank N, Hodge MR, et al (2004) T follicular helper cells express a distinctive transcriptional profile, reflecting their role as non-Th1/Th2 effector cells that provide help for B cells. J Immunol 173: 68–78. 15210760 10.4049/jimmunol.173.1.68

[16] Nurieva RI, Chung Y, Hwang D, Yang XO, Kang HS, et al (2008) Generation of T follicular helper cells is mediated by interleukin-21 but independent of T helper 1, 2, or 17 cell lineages. Immunity 29: 138–149. 10.1016/j.immuni.2008.05.009 18599325 PMC2556461

[17] Rasheed AU, Rahn HP, Sallusto F, Lipp M, Muller G (2006) Follicular B helper T cell activity is confined to CXCR5(hi)ICOS(hi) CD4 T cells and is independent of CD57 expression. Eur J Immunol 36: 1892–1903. 16791882 10.1002/eji.200636136

[18] Vogelzang A, McGuire HM, Yu D, Sprent J, Mackay CR, et al (2008) A fundamental role for interleukin-21 in the generation of T follicular helper cells. Immunity 29: 127–137. 10.1016/j.immuni.2008.06.001 18602282

[19] Bessa J, Kopf M, Bachmann MF (2010) Cutting edge: IL-21 and TLR signaling regulate germinal center responses in a B cell-intrinsic manner. J Immunol 184: 4615–4619. 10.4049/jimmunol.0903949 20368279

[20] Linterman MA, Beaton L, Yu D, Ramiscal RR, Srivastava M, et al (2010) IL-21 acts directly on B cells to regulate Bcl-6 expression and germinal center responses. J Exp Med 207: 353–363. 10.1084/jem.20091738 20142429 PMC2822609

[21] Zotos D, Coquet JM, Zhang Y, Light A, D'Costa K, et al (2010) IL-21 regulates germinal center B cell differentiation and proliferation through a B cell-intrinsic mechanism. J Exp Med 207: 365–378. 10.1084/jem.20091777 20142430 PMC2822601

[22] Jin H, Carrio R, Yu A, Malek TR (2004) Distinct activation signals determine whether IL-21 induces B cell costimulation, growth arrest, or Bim-dependent apoptosis. J Immunol 173: 657–665. 15210829 10.4049/jimmunol.173.1.657

[23] Rasheed MA, Latner DR, Aubert RD, Gourley T, Spolski R, et al (2013) Interleukin-21 is a critical cytokine for the generation of virus-specific long-lived plasma cells. J Virol 87: 7737–7746. 10.1128/JVI.00063-13 23637417 PMC3700268

[24] Zeng R, Spolski R, Casas E, Zhu W, Levy DE, et al (2007) The molecular basis of IL-21-mediated proliferation. Blood 109: 4135–4142. 17234735 10.1182/blood-2006-10-054973 PMC1885510

[25] Konforte D, Paige CJ (2006) Identification of cellular intermediates and molecular pathways induced by IL-21 in human B cells. J Immunol 177: 8381–8392. 17142735 10.4049/jimmunol.177.12.8381

[26] Fornek JL, Tygrett LT, Waldschmidt TJ, Poli V, Rickert RC, et al (2006) Critical role for Stat3 in T-dependent terminal differentiation of IgG B cells. Blood 107: 1085–1091. 16223771 10.1182/blood-2005-07-2871 PMC1895906

[27] Ozaki K, Spolski R, Ettinger R, Kim HP, Wang G, et al (2004) Regulation of B cell differentiation and plasma cell generation by IL-21, a novel inducer of Blimp-1 and Bcl-6. J Immunol 173: 5361–5371. 15494482 10.4049/jimmunol.173.9.5361

[28] Arguni E, Arima M, Tsuruoka N, Sakamoto A, Hatano M, et al (2006) JunD/AP-1 and STAT3 are the major enhancer molecules for high Bcl6 expression in germinal center B cells. Int Immunol 18: 1079–1089. 16702165 10.1093/intimm/dxl041

[29] Dent AL, Shaffer AL, Yu X, Allman D, Staudt LM (1997) Control of inflammation, cytokine expression, and germinal center formation by BCL-6. Science 276: 589–592. 9110977 10.1126/science.276.5312.589

[30] Liang X, Collins CM, Mendel JB, Iwakoshi NN, Speck SH (2009) Gammaherpesvirus-driven plasma cell differentiation regulates virus reactivation from latently infected B lymphocytes. PLoS Pathog 5: e1000677 10.1371/journal.ppat.1000677 19956661 PMC2777334

[31] Wilson SJ, Tsao EH, Webb BL, Ye H, Dalton-Griffin L, et al (2007) X box binding protein XBP-1s transactivates the Kaposi's sarcoma-associated herpesvirus (KSHV) ORF50 promoter, linking plasma cell differentiation to KSHV reactivation from latency. J Virol 81: 13578–13586. 17928342 10.1128/JVI.01663-07 PMC2168861

[32] Laichalk LL, Thorley-Lawson DA (2005) Terminal differentiation into plasma cells initiates the replicative cycle of Epstein-Barr virus in vivo. J Virol 79: 1296–1307. 15613356 10.1128/JVI.79.2.1296-1307.2005 PMC538585

[33] Sun CC, Thorley-Lawson DA (2007) Plasma cell-specific transcription factor XBP-1s binds to and transactivates the Epstein-Barr virus BZLF1 promoter. J Virol 81: 13566–13577. 17898050 10.1128/JVI.01055-07 PMC2168822

[34] Yu F, Feng J, Harada JN, Chanda SK, Kenney SC, et al (2007) B cell terminal differentiation factor XBP-1 induces reactivation of Kaposi's sarcoma-associated herpesvirus. FEBS Lett 581: 3485–3488. 17617410 10.1016/j.febslet.2007.06.056

[35] Bhende PM, Dickerson SJ, Sun X, Feng WH, Kenney SC (2007) X-box-binding protein 1 activates lytic Epstein-Barr virus gene expression in combination with protein kinase D. J Virol 81: 7363–7370. 17494074 10.1128/JVI.00154-07 PMC1933364

[36] Decalf J, Godinho-Silva C, Fontinha D, Marques S, Simas JP (2014) Establishment of murine gammaherpesvirus latency in B cells is not a stochastic event. PLoS Pathog 10: e1004269 10.1371/journal.ppat.1004269 25079788 PMC4117635

[37] Victora GD, Schwickert TA, Fooksman DR, Kamphorst AO, Meyer-Hermann M, et al (2010) Germinal center dynamics revealed by multiphoton microscopy with a photoactivatable fluorescent reporter. Cell 143: 592–605. 10.1016/j.cell.2010.10.032 21074050 PMC3035939

[38] Schwickert TA, Lindquist RL, Shakhar G, Livshits G, Skokos D, et al (2007) In vivo imaging of germinal centres reveals a dynamic open structure. Nature 446: 83–87. 17268470 10.1038/nature05573

[39] Suzuki K, Grigorova I, Phan TG, Kelly LM, Cyster JG (2009) Visualizing B cell capture of cognate antigen from follicular dendritic cells. J Exp Med 206: 1485–1493. 10.1084/jem.20090209 19506051 PMC2715076

[40] Konforte D, Paige CJ (2009) Interleukin-21 regulates expression of the immediate-early lytic cycle genes and proteins in Epstein-Barr Virus infected B cells. Virus Res 144: 339–343. 10.1016/j.virusres.2009.05.003 19447148

[41] Konforte D, Simard N, Paige CJ (2008) Interleukin-21 regulates expression of key Epstein-Barr virus oncoproteins, EBNA2 and LMP1, in infected human B cells. Virology 374: 100–113. 10.1016/j.virol.2007.12.027 18222514

[42] Kis LL, Salamon D, Persson EK, Nagy N, Scheeren FA, et al (2010) IL-21 imposes a type II EBV gene expression on type III and type I B cells by the repression of C- and activation of LMP-1-promoter. Proc Natl Acad Sci U S A 107: 872–877. 10.1073/pnas.0912920107 20080768 PMC2818931

[43] Nagy N, Adori M, Rasul A, Heuts F, Salamon D, et al (2012) Soluble factors produced by activated CD4+ T cells modulate EBV latency. Proc Natl Acad Sci U S A 109: 1512–1517. 10.1073/pnas.1120587109 22307606 PMC3277165

[44] Ozaki K, Spolski R, Feng CG, Qi CF, Cheng J, et al (2002) A critical role for IL-21 in regulating immunoglobulin production. Science 298: 1630–1634. 12446913 10.1126/science.1077002

[45] Weck KE, Kim SS, Virgin HI, Speck SH (1999) B cells regulate murine gammaherpesvirus 68 latency. J Virol 73: 4651–4661. 10233924 10.1128/jvi.73.6.4651-4661.1999 PMC112506

[46] Weck KE, Barkon ML, Yoo LI, Speck SH, Virgin HI (1996) Mature B cells are required for acute splenic infection, but not for establishment of latency, by murine gammaherpesvirus 68. J Virol 70: 6775–6780. 8794315 10.1128/jvi.70.10.6775-6780.1996 PMC190721

