# Correction: Interleukin 21 Signaling in B Cells Is Required for Efficient Establishment of Murine Gammaherpesvirus Latency

**DOI:** 10.1371/journal.ppat.1004922

**Published:** 2015-05-13

**Authors:** The PLOS Pathogens Staff

There is an error in Table 1, which was introduced during the typesetting process. The publisher apologizes for this error. The data given for day 20, Total YFP+ PCs (SEM) in the IL21R-/- mice reads: “3)”. It should read: “448 (83)”. Please see the corrected table here.

**Table 1:** Average number of YFP^+^ cells per spleen.

	Total YFP+ cells (SEM)^a^		Total YFP+ GC B cells (SEM)^b^		Total YFP+ PCs (SEM)^c^	
	C57Bl6	Il21R^-/-^	C57Bl6	Il21R^-/-^	C57Bl6	Il21R^-/-^
d14	21,129 (6,898)	6,012 (1,256)	14,360 (4998)	2,515 (644)	4,034 (1,398)	1,127 (264)
d16	313,528 (117,143)	17,966 (3,414)	273,788 (110,482)	9,772 (1,929)	37,081 (13,599)	1,823 (327)
d18	254,735 (72,400)	20,579 (5,316)	200,146 (56,323)	14,089 (4170)	29,466 (7,862)	1,088 (248)
d20	84,718 (25,889)	9,637 (2,317)	65,518 (20,896)	4,648 (1,019)	7,641 (2,145)	448 (83)

^a.^ Calculated from mice infected in Fig 1A and 1B.

^b.^ Calculated from mice infected in Fig 5A and 5B.

^c.^ Calculated from mice infected in Fig 2C and 2D.
